# Prognostic value of the combination of neutrophil-to-lymphocyte ratio, monocyte-to-lymphocyte ratio and platelet-to-lymphocyte ratio on mortality in patients on maintenance hemodialysis

**DOI:** 10.1186/s12882-022-03020-1

**Published:** 2022-12-08

**Authors:** Jiaxian Liao, Dongyan Wei, Chenghui Sun, Yuqi Yang, Yinxia Wei, Xinhui Liu

**Affiliations:** 1 Department of Nephrology, Hechi Traditional Chinese Medicine Hospital, Hechi, Guangxi China; 2 Department of Nephrology, Shenzhen Traditional Chinese Medicine Hospital, Guangzhou University of Chinese Medicine, Shenzhen, Guangdong China

**Keywords:** Hemodialysis, Neutrophil-to-lymphocyte ratio, Monocyte-to-lymphocyte ratio, Platelet-to-lymphocyte ratio, The inflammation score, Mortality

## Abstract

**Background:**

Hemodialysis (HD) is the most important renal replacement therapy for patients with end-stage kidney disease (ESKD). Systemic inflammation is a risk factor of mortality in HD patients. Neutrophil-to-lymphocyte ratio (NLR), monocyte-to-lymphocyte ratio (MLR), and platelet-to-lymphocyte ratio (PLR) are new inflammatory markers. However, previous studies have inconsistent conclusions about the predictive value of NLR, MLR and PLR on mortality of HD patients. The aim of this study was to establish an inflammation scoring system by including NLR, MLR and PLR, and evaluate the association between the inflammation score and all-cause and cardiovascular mortality in HD patients.

**Methods:**

In this single center retrospective cohort study, 213 incident HD patients from January 1, 2015 to December 31, 2020 were included. Baseline demographic and clinical data and laboratory measurements were collected. According to the optimal cut-off values, NLR, MLR and PLR were assigned 0 or 1 point, respectively. Then, the inflammation score was obtained by adding the NLR, MLR and PLR scores. All patients were followed until July 31, 2021. The associations of the inflammation score with all-cause and cardiovascular mortality were assessed by multivariable-adjusted Cox models.

**Results:**

Of 213 patients, the mean (± SD) age was 56.8 ± 14.4 years, 66.2% were men, and 32.9% with diabetes. The primary cause of ESKD was mainly chronic glomerulonephritis (46.5%) and diabetic nephropathy (28.6%). The median inflammation score was 2 (interquartile range = 1–3). During a median 30 months (interquartile range = 17–50 months) follow-up period, 53 patients had died, of which 33 deaths were caused by cardiovascular disease. After adjusting for demographics, primary diseases and other confounders in multivariable model, the inflammation score = 3 was associated with a hazard ratio for all-cause mortality of 4.562 (95% confidence interval, 1.342–15.504, *P* = 0.015) and a hazard ratio for cardiovascular mortality of 4.027 (95% confidence interval, 0.882–18.384, *P* = 0.072).

**Conclusion:**

In conclusion, an inflammation scoring system was established by including NLR, MLR and PLR, and the higher inflammation score was independently associated with all-cause mortality in HD patients.

**Supplementary Information:**

The online version contains supplementary material available at 10.1186/s12882-022-03020-1.

## Background

Chronic kidney disease (CKD) is a global public health problem. With the decline in renal function, patients with end-stage kidney disease (ESKD) can choose hemodialysis (HD), peritoneal dialysis (PD) or kidney transplantation as renal replacement therapy to improve their quality of life. Globally, HD is still the most important renal replacement therapy. With the continuous development and optimization of dialysis technology, the survival time of maintenance hemodialysis (MHD) patients is gradually prolonged. However, even in developed countries, the mortality rate of MHD patients remains high. According to data from the United States Renal Data System (USRDS) in 2019, the MHD mortality rate in 2017 was 167 per 1,000 patient-years [1]. Studies have shown that 30-50% of MHD patients have chronic inflammation state [2]. Persistent inflammation can lead to a variety of complications, especially cardiovascular disease, malnutrition and anemia, thereby increasing the mortality of MHD patients [3]. Although less than 5% of MHD patients die directly from inflammation, inflammation can interact with a variety of risk factors and has an important impact on the prognosis of MHD patients. Studies have found that inflammation is closely related to high-risk factors of death such as myocardial hypertrophy, ventricular dysfunction, atherosclerosis, protein energy consumption, anemia and renal bone disease [4–6]. Therefore, the development of inflammation-related indicators has important clinical value for judging the prognosis of MHD patients and guiding early intervention.

Recent studies have found that the ratios of different blood cell components are new inflammatory markers, which have good predictive value in the outcome of CKD, cardiovascular disease, rheumatic disease, etc., including neutrophil-to-lymphocyte ratio (NLR) [7–10], monocyte-to-lymphocyte ratio (MLR) [11–14], and platelet-to-lymphocyte ratio (PLR) [15–19]. Previous studies have inconsistent conclusions about the predictive value of NLR, MLR and PLR on mortality of HD patients. Xiang et al. suggested that higher MLR was a strong and independent predictor of all-cause and cardiovascular mortality and overwhelmed NLR among HD patients [20]. Another study found that both NLR and PLR were associated with all-cause mortality in prevalent HD patients, but only PLR could independently predict all-cause mortality in these populations [21]. We hypothesized that the combination of NLR, MLR and PLR could form a new indicator for predicting the mortality of HD patients. Therefore, we incorporated NLR, MLR, and PLR into an inflammation scoring system and investigated the value of inflammation score in predicting all-cause and cardiovascular mortality in HD patients.

## Methods

### Study population

We included all incident HD patients from Department of Nephrology, Hechi Traditional Chinese Medicine Hospital from January 1, 2015, to December 31, 2020. Patients who were younger than 18 years, started HD in other hospitals, transferred from PD or failed renal transplantation, and undergone HD for less than 3 months were excluded in this study. The study was conducted in compliance with the ethical principles of the Helsinki Declaration and approved by the Ethics Committees of Hechi Traditional Chinese Medicine Hospital. All participants have provided written informed consent.

### Data collection

All baseline data were collected at 1–3 months after HD initiation. Demographic data included age, sex, primary cause of ESKD, and history of diabetes. Clinical and biochemical data included body mass index (BMI), blood pressure, residual urine volume, neutrophils, lymphocytes, monocytes, platelets, hemoglobin, serum albumin, serum creatinine, blood urea nitrogen, serum uric acid, calcium, phosphorus, intact parathyroid hormone (iPTH), C-reactive protein (CRP), total cholesterol, triglycerides, low-density lipoprotein cholesterol (LDL-C), high-density lipoprotein cholesterol (HDL-C), alanine transaminase and total bilirubin. NLR, MLR and PLR were calculated by dividing neutrophils, monocytes, and platelets by lymphocytes, respectively.

### Outcomes

The primary outcome of the present study was all-cause mortality, and the secondary outcome was cardiovascular mortality. Cardiovascular death was defined as death due to coronary events, arrhythmias, sudden cardiac death, congestive heart failure or cerebrovascular events [22]. All patients were followed up until death, cessation of HD or on July 31, 2021.

### Statistical analysis

**Fig. 1:**
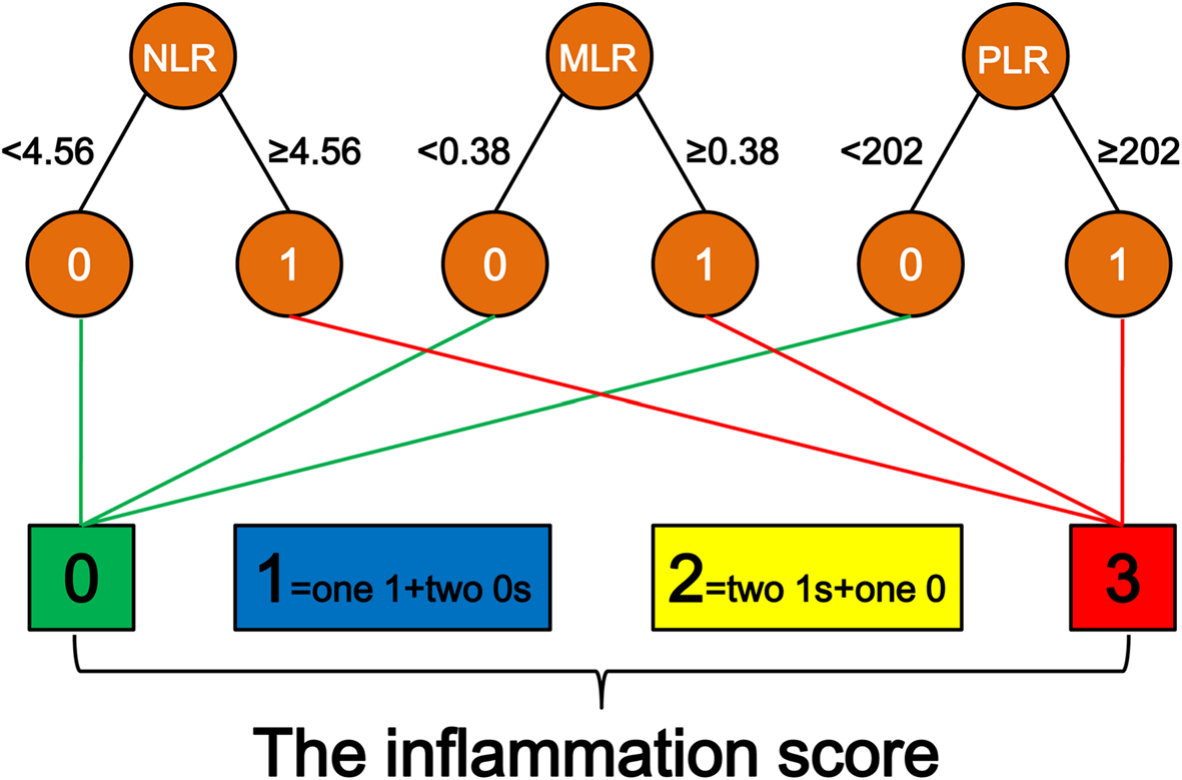
Flow chart illustrating how the inflammation score was calculated

The inflammation score established in this study was determined by the values of NLR, MLR and PLR. First, we determined the cut-off values of NLR, MLR and PLR through receiver operating characteristic (ROC) analyses. We found an NLR cut-off value of 4.56 had a sensitivity of 0.695 and a specificity of 0.602; a MLR cut-off value of 0.38 had a sensitivity of 0.780 and a specificity of 0.634; a PLR cut-off value of 202 had a sensitivity of 0.559 and a specificity of 0.509 for differentiating all-cause mortality, via ROC analyses. According to the optimal cut-off values, NLR, MLR and PLR were assigned 0 or 1 point, respectively. Then, the inflammation score was obtained by adding the NLR, MLR and PLR scores (Fig. 1). Based on the inflammation score, all participants were divided into four groups. The data were presented as frequency (percentage) for categorical variables, mean ± standard deviation for normally distributed continuous variables and median (inter-quartile range) for skewed continuous variables. Comparison among groups was performed using χ^2^ test, One-way ANOVA test or Kruskal-Wallis test in categorical or continuous variables, respectively. Survival probabilities were estimated from Kaplan–Meier curves followed by log-rank test to assess differences among groups. The univariate and multivariate Cox proportional hazards models were used to examine the associations between inflammation score and all-cause and cardiovascular mortality. The multivariate Cox regression models were constructed by including eligible covariates with *P* values < 0.05 in the univariate Cox analyses or for importance of clinical concern. The results were expressed as the hazard ratio (HR) and 95% confidence interval (CI). All statistical analyses were conducted by using SPSS software version 16.0 (SPSS Inc., Chicago, IL, USA). A value of *P* < 0.05 was considered statistically significant.

## Results

### Patient characteristics

A total of 213 maintenance hemodialysis (MHD) patients were enrolled in this study. The mean (± SD) age was 56.8 ± 14.4 years, 66.2% were men, and 32.9% with diabetes. The primary cause of ESKD was mainly chronic glomerulonephritis (46.5%) and diabetic nephropathy (28.6%). Baseline characteristics of the patients stratified by the inflammation score were shown in Table 1. Not surprisingly, patients with higher inflammation score had higher levels of NLR, MLR, PLR and CRP (*P* < 0.001). In addition to these variables, 24 h urine output, blood urea nitrogen and LDL-C were also statistically different in the comparison among groups (*P* < 0.05). There were no significant differences among groups in age, BMI, blood pressure, hemoglobin and albumin (Table 1).

**Table 1:** Baseline characteristics of participants stratified by the inflammation score

Variable	The inflammation score				*P* value
	0 (*n* = 45)	1 (*n* = 33)	2 (*n* = 41)	3 (*n* = 94)	
Male (%)	30 (66.7)	18 (54.5)	31 (75.6)	62 (66.0)	0.304
Age (y)	52.8 ± 14.9	58.2 ± 11.9	57.3 ± 16.7	58.0 ± 13.8	0.210
Body mass index (kg/m^2^)	22.8 ± 3.5	23.1 ± 3.7	22.8 ± 3.3	23.2 ± 3.4	0.888
Diabetes (%)	12 (26.7)	12 (36.4)	10 (24.4)	36 (38.3%)	0.313
24 h urine output (mL)	650 (350–1075)	850 (550–1275)	525 (300–750)	725 (400–1035)	**0.011**
Systolic pressure (mmHg)	165 ± 28	162 ± 29	165 ± 26	162 ± 29	0.900
Diastolic pressure (mmHg)	96 ± 19	93 ± 19	96 ± 16	96 ± 18	0.780
Mean arterial pressure (mmHg)	119 ± 20	116 ± 21	119 ± 18	118 ± 21	0.849
NLR	3.05 (2.37–3.66)	3.71 (3.11–4.52)	5.92 (4.91–8.40)	10.73 (7.18–16.15)	**< 0.001**
MLR	0.29 (0.22–0.33)	0.40 (0.34–0.51)	0.46 (0.38–0.65)	0.67 (0.50–0.93)	**< 0.001**
PLR	124 (86–147)	165 (119–197)	194 (156–262)	286 (236–391)	**< 0.001**
Hemoglobin (g/L)	77.9 ± 19.5	84.3 ± 14.6	81.9 ± 20.6	80.1 ± 22.1	0.556
Albumin (g/L)	33.7 ± 8.2	36.5 ± 5.7	34.2 ± 6.3	35.3 ± 5.8	0.203
Blood urea nitrogen (mmol/L)	28.1 ± 10.3	28.1 ± 10.7	30.3 ± 14.2	33.8 ± 13.3	**0.030**
Serum creatinine (µmol/L)	801 (643–1273)	825 (667–1187)	847 (651–1102)	856 (656–1202)	0.955
Serum uric acid (µmol/L)	512 ± 142	526 ± 139	529 ± 144	548 ± 170	0.623
Calcium (mmol/L)	1.91 ± 0.28	1.97 ± 0.29	1.87 ± 0.31	1.91 ± 0.30	0.485
Phosphorus (mmol/L)	1.82 ± 0.68	1.80 ± 0.63	1.84 ± 0.70	1.90 ± 0.75	0.881
iPTH (pg/mL)	161 (87–292)	186 (115–382)	255 (163–411)	248 (97–414)	0.136
CRP (mg/L)	1.60 (0.00-5.15)	3.30 (0.10–9.65)	7.90 (3.00-31.65)	18.80 (2.00–46.00)	**< 0.001**
ALT (U/L)	17 (11–24)	15 (10–19)	19 (12–26)	16 (9–22)	0.397
Total bilirubin (mmol/L)	4.40 (2.93–6.93)	5.40 (3.15–6.75)	4.90 (3.85–7.95)	4.90 (3.10–6.60)	0.609
Total cholesterol (mmol/L)	5.06 ± 1.89	4.53 ± 1.39	4.65 ± 1.48	4.40 ± 1.17	0.100
Triglyceride (mmol/L)	1.33 (0.95–2.01)	1.53 (0.98–2.36)	1.17 (0.99–1.63)	1.22 (0.89–1.86)	0.249
LDL-C (mmol/L)	2.86 ± 1.71	2.61 ± 1.18	2.67 ± 1.06	2.28 ± 0.83	**0.039**
HDL-C (mmol/L)	1.27 ± 0.31	1.18 ± 0.39	1.27 ± 0.40	1.20 ± 0.46	0.617

*Abbreviations*: *ALT* alanine aminotransferase, *CRP* C-reactive protein, *HDL-C* high-density lipoprotein cholesterol, *iPTH* parathyroid hormone, *LDL-C* low-density lipoprotein cholesterol, *MLR* monocyte-to-lymphocyte ratio, *NLR* neutrophil-to-lymphocyte ratio, *PLR* platelet-to-lymphocyte ratio

### The inflammation score and all-cause and Cardiovascular Mortality

**Fig. 2:**
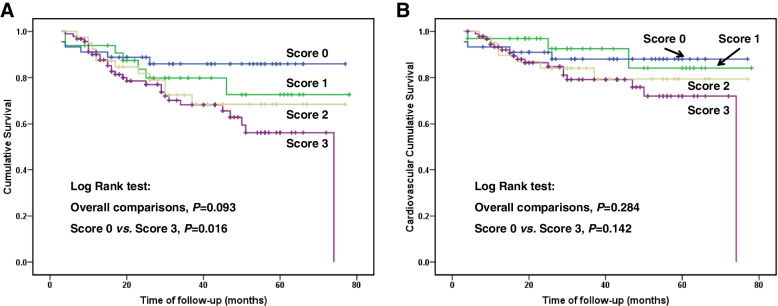
Patient survival curves for those with different inflammation score: all-cause mortality (**A**) and cardiovascular mortality (**B**)

The median follow-up period was 30 months (inter-quartile range 17 to 50 months). By the end of the study, 53 patients (24.9%) had died, of which 33 deaths (62.3%) were caused by cardiovascular disease. Kaplan-Meier analyses found that the mean survival time for all-cause mortality was 67.7 months in score 0 group, 63.2 months in score 1 group, 59.3 months in score 2 group, and 53.2 months in score 3 group. In Log Rank test, the *P* value of overall comparisons among groups was 0.093, the comparison between score 0 group and score 3 group was statistically significant. (*P* = 0.016, Fig. 2A). The mean survival time for cardiovascular mortality was 69.1 months in score 0 group, 70.7 months in score 1 group, 65.1 months in score 2 group, and 60.5 months in score 3 group. Patient in score 3 group had the lowest cardiovascular survival rate among the groups, although not statistically significant (Fig. 2B).

Associations between the inflammation score and all-cause and cardiovascular mortality were summarized in Table 2. Regardless of the adjustment methods used, there was significant association between the inflammation score = 3 and all-cause mortality (*P* < 0.05), and *P* values for trend were less than 0.05 in all models. In terms of cardiovascular mortality, the risk of death also tended to increase as the inflammation score increases. In model 4, which was a maximally adjusted model including age, sex, body mass index, primary cause of ESKD, mean arterial pressure, 24 h urine output, hemoglobin, albumin, calcium, phosphorus, iPTH, total cholesterol, and triglyceride, HRs for all-cause and cardiovascular mortality were 4.562 (95% CI: 1.342–15.504, *P* = 0.015) and 4.027 (95% CI: 0.882–18.384, *P* = 0.072) respectively, when the inflammation score increased from 0 to 3 (Table 2). CRP is a traditional marker of inflammation. To compare the predictive value of the new inflammation score and CRP for mortality in HD patients in this study, we calculated the HRs of CRP for all-cause and cardiovascular mortality in the same Cox model as in Table 2. The results showed that CRP was not significantly associated with all-cause and cardiovascular mortality in our study, either in univariate or multivariate models (Supplementary Table S1).

**Table 2:** Associations between the inflammation score and all-cause and cardiovascular mortality

	Model 1^a^		Model 2^b^		Model 3^c^		Model 4^d^	
	HR (95% CI)	*P* value	HR (95% CI)	*P* value	HR (95% CI)	*P* value	HR (95% CI)	*P* value
All-cause mortality								
The inflammation score = 0	Reference		Reference		Reference		Reference	
The inflammation score = 1	1.601 (0.537-4.774)	0.398	1.573 (0.523-4.732)	0.420	1.930 (0.604-6.162)	0.267	2.825 (0.707-11.285)	0.142
The inflammation score = 2	2.148 (0.794-5.814)	0.132	2.018 (0.739-5.508)	0.170	2.067 (0.704-6.068)	0.186	3.300 (0.882-12.344)	0.076
The inflammation score = 3	2.781 (1.153-6.708)	**0.023**	2.640 (1.085-6.425)	**0.032**	2.969 (1.115-7.909)	**0.029**	4.562 (1.342-15.504)	**0.015**
*P* for trend	**0.013**		**0.020**		**0.024**		**0.010**	
Cardiovascular mortality								
The inflammation score = 0	Reference		Reference		Reference		Reference	
The inflammation score = 1	0.801 (0.191-3.366)	0.762	0.775 (0.182-3.292)	0.730	1.029 (0.225-4.694)	0.971	2.033 (0.324-12.764)	0.449
The inflammation score = 2	1.629 (0.516-5.140)	0.405	1.580 (0.496-5.038)	0.439	1.655 (0.475-5.769)	0.429	3.178 (0.619-16.302)	0.166
The inflammation score = 3	2.049 (0.759-5.529)	0.157	1.979 (0.724-5.414)	0.184	2.200 (0.708-6.835)	0.173	4.027 (0.882-18.384)	0.072
*P* for trend	0.076		0.087		0.110		**0.047**	

^a^Model 1: unadjusted

^b^Model 2: adjusted for age and sex

^c^Model 3: adjusted for model 2 covariates and body mass index, primary cause of ESKD, mean arterial pressure, and 24 h urine output

^d^Model 4: adjusted for model 3 covariates and hemoglobin, albumin, calcium, phosphorus, iPTH, total cholesterol, and triglyceride

*Abbreviations*: *HR* hazard ratio, *95% CI* 95% confidence interval

## Discussion

In the present study, we established an inflammation scoring system by including NLR, MLR and PLR. We found that higher inflammation score was independently associated with all-cause mortality in HD patients.

Chronic inflammation is prevalent in patients with CKD and contributes to morbidity and mortality among dialysis patients [23]. CRP is a traditional marker of inflammation, and has been reported to predict all-cause and cardiovascular mortality in HD patients [3, 24]. However, inflammation, especially non-infectious inflammation, induces significant individual differences in CRP levels [25]. In the present study, we did not find significant association between CRP and all-cause and cardiovascular mortality (Supplementary Table S1). NLR, MLR and PLR have recently been used as indicators of inflammation. NLR has a greater predictability than total white blood cell count or neutrophil count in cardiovascular disease [8]. Neuen et al. analyzed 170 HD patients with a median follow-up of 37 months and found that NLR was independently associated with both all-cause and cardiovascular mortality [26]. Another study included 268 HD patients and revealed that high NLR was an independent predictor of all-cause and cardiovascular mortality when adjusted for other risk factors [27]. Besides neutrophil and lymphocytes, monocytes play a key role in inflammation. A previous study pointed the contribution of circulating leukocytes, particularly granulocytes and monocytes to the pathogenesis of ESKD-induced oxidative stress and its exacerbation by HD procedure [28]. One study compared the predictive values of MLR and NLR for mortality in HD patients. The results demonstrated that MLR was a strong predictor of all-cause and cardiovascular mortality among HD patients. However, NLR was not independently associated with mortality in multivariate Cox models [20]. Platelets release pro-inflammatory mediators, such as chemokines and cytokines [29]. The PLR is also an inflammatory marker, which may be used in many diseases for predicting inflammation and mortality [30]. Yaprak et al. included 80 prevalent HD patients and found that both NLR and PLR were associated with all-cause mortality, but only PLR could independently predict all-cause mortality in these populations [21]. The possible reason for the inconsistent results of above studies is that the focus of NLR, MLR and PLR in reflecting the inflammatory state is different. Although NLR, MLR and PLR have certain predictive value for the prognosis of HD patients, their predictive power varies in different study cohorts. Therefore, the combination of NLR, MLR and PLR can theoretically reflect the inflammatory state of the body more comprehensively, thereby more accurately predicting the prognosis of HD patients. The present study verified this hypothesis. As the inflammation score, determined by NLR, MLR and PLR, increases, the risk of all-cause and cardiovascular death in HD patients increases (Table 2). NLR, MLR and PLR can simply be calculated from full blood count, which is less costly compared with other inflammatory markers such as interleukins, tumor necrosis factor-alpha and CRP. Hence, the inflammation score appears to be a cost-effective index with clinical predictability and prognostication in HD patients.

There are several limitations of the present study. First, it was a single center study, which may affect its external validity. Second, it was a retrospective study, thus the results indicated associations but not causal relationships. Third, due to the relatively small sample size, the conclusions of the study need to be confirmed in larger patient populations. Fourth, we only included the baseline data instead of all the data during the follow-up period, which may cause bias in the results.

## Conclusion

In conclusion, we established an inflammation scoring system by combination of NLR, MLR and PLR. We found an independent relationship between higher inflammation score and all-cause mortality in HD patients. This result suggests that the inflammation score may be a tool for clinicians to judge the prognosis of HD patients.

## Supplementary Information

**Additional file 1: Supplementary Table S1.** Associations between C-reactive protein and all-cause and cardiovascular mortality. 

## Data Availability

The datasets used and analysed during the current study available from the corresponding author on reasonable request.

## Abbreviations

ANOVA: Analysis of variance
BMI: Body mass index
CKD: Chronic kidney disease
CI: Confidence interval
CRP: C-reactive protein
ESKD: End-stage kidney disease
HD: Hemodialysis
HDL-C: High-density lipoprotein cholesterol
HR: Hazard ratio
iPTH: Intact parathyroid hormone
LDL-C: Low-density lipoprotein cholesterol
MHD: Maintenance hemodialysis
MLR: Monocyte-to-lymphocyte ratio
NLR: Neutrophil-to-lymphocyte ratio
PD: Peritoneal dialysis
PLR: Platelet-to-lymphocyte ratio
ROC: Receiver operating characteristic
SD: Standard deviation
USRDS: United States Renal Data System
