# Supplementary material for: Prognostic value of the combination of neutrophil-to-lymphocyte ratio, monocyte-to-lymphocyte ratio and platelet-to-lymphocyte ratio on mortality in patients on maintenance hemodialysis
Source: BMC Nephrol. 2022 Dec 8;23:393. doi: 10.1186/s12882-022-03020-1 (PMC9730573; doi:10.1186/s12882-022-03020-1)
Supplement: Supplementary file 1 — Additional file 1: Supplementary Table S1. Associations between C-reactive protein and all-cause and cardiovascular mortality. [file 12882_2022_3020_MOESM1_ESM.docx]

**Supplementary Table S1.** Associations between C-reactive protein and all-cause and cardiovascular mortality

|  | Model 1^a^ | | Model 2^b^ | | Model 3^c^ | | Model 4^d^ | |
| --- | --- | --- | --- | --- | --- | --- | --- | --- |
|  | HR (95% CI) | *P* value | HR (95% CI) | *P* value | HR (95% CI) | *P* value | HR (95% CI) | *P* value |
| All-cause mortality |  |  |  |  |  |  |  |  |
| CRP (per mg/L) | 1.002 (0.994-1.010) | 0.631 | 1.000 (0.992-1.009) | 0.420 | 1.000 (0.991-1.009) | 0.992 | 1.000 (0.990-1.009) | 0.942 |
| Cardiovascular mortality |  |  |  |  |  |  |  |  |
| CRP (per mg/L) | 1.000 (0.989-1.012) | 0.961 | 0.999 (0.988-1.011) | 0.930 | 0.998 (0.986-1.011) | 0.809 | 0.996 (0.982-1.010) | 0.572 |

^a^ Model 1: unadjusted.

^b^ Model 2: adjusted for age and gender.

^c^ Model 3: adjusted for model 2 covariates and body mass index, primary cause of ESKD, mean arterial pressure, and 24h urine output.

^d^ Model 4: adjusted for model 3 covariates and hemoglobin, albumin, calcium, phosphorus, iPTH, total cholesterol, and triglyceride.

Abbreviations: CRP, C-reactive protein; HR, hazard ratio; 95% CI, 95% confidence interval.
